# KmerCrypt: private k-mer search with homomorphic encryption

**DOI:** 10.1093/bib/bbaf648

**Published:** 2025-12-08

**Authors:** Kimonas Provatas, Ioannis Mouratidis, Ilias Georgakopoulos-Soares

**Affiliations:** Division of Pharmacology and Toxicology, College of Pharmacy, The University of Texas at Austin, Dell Paediatric Research Institute, 1400 Barbara Jordan Blvd, Austin, TX 78723, United States; Division of Pharmacology and Toxicology, College of Pharmacy, The University of Texas at Austin, Dell Paediatric Research Institute, 1400 Barbara Jordan Blvd, Austin, TX 78723, United States; Division of Pharmacology and Toxicology, College of Pharmacy, The University of Texas at Austin, Dell Paediatric Research Institute, 1400 Barbara Jordan Blvd, Austin, TX 78723, United States

**Keywords:** homomorphic encryption, k-mer search, genomic privacy, secure computation, cloud bioinformatics

## Abstract

Outsourcing the storage and analysis of genomic data to third-party servers is often necessary due to the scale of modern datasets, but it introduces significant privacy challenges that must be addressed to ensure secure handling. K-mer-based analyses offer broad applications across genomics research, clinical diagnostics, pathogen surveillance, and metagenomic classification, though implementation requires careful ethical and technical considerations, particularly when processing human genomic data in clinical settings. We present a novel protocol utilizing homomorphic encryption that enables a client to store a fully encrypted version of a genome on an untrusted server and perform private k-mer searches. The protocol ensures the server never gains access to the client’s non-encrypted genome sequence, nor does it learn the content of any k-mer query. After a one-time client-side encryption of the genome, the server performs all computations on ciphertext, returning only encrypted results that can be decrypted solely by the data owner. This framework transforms an honest but curious cloud server into a secure storage and computation system, enabling practical and confidential querying of encrypted, client-owned genomic data. The system supports exact k-mer searches on genomic data, as well as position weight matrix searches. Finally, we provide KmerCrypt, a private k-mer search toolkit that implements this protocol, offering researchers an efficient and secure solution for querying encrypted genomic datasets without compromising privacy.

## Introduction

The exponential growth of genomic sequencing technologies has ushered in an era of unprecedented data generation. Public repositories such as NCBI RefSeq and GenBank now host millions of genome assemblies [1, 2], and databases such as gnomAD store germline polymorphisms for hundreds of thousands of individuals [3]. At the same time, individual laboratories and healthcare institutions routinely generate terabytes of genomic data. This deluge of information has immense potential for advancing precision medicine, clinical genomics, and biotechnology. However, the computational burden of processing such vast datasets has outpaced the capabilities of many local infrastructures, prompting a shift toward leveraging cloud computing for storage and analysis [4–6].

Despite its scalability and cost-effectiveness, outsourcing genomic computation to third-party cloud providers poses significant privacy risks. Personal genomes and genomic data are among the most sensitive forms of identifiable data, and their misuse could have profound ethical, legal, and social implications [7, 8]. Regulatory frameworks such as HIPAA in the USA, GDPR in the European Union, and other genomic privacy laws impose strict limitations on the use and dissemination of genomic information, often hindering the adoption of cloud-based solutions in clinical and research settings [9]. To address this dual demand for scalability and privacy, there is an urgent need for computational frameworks that allow secure outsourcing of genomic analysis while guaranteeing data confidentiality. Such frameworks should enable data owners to delegate computation to untrusted servers without exposing their genomic sequences or analytic intent [8].

A k-mer is a substring of length k extracted from a longer nucleotide or amino acid sequence. K-mer searching is an integral component across many bioinformatics pipelines and applications, including genome assembly, sequence alignment, metagenomic profiling, variant detection, evolutionary analysis, and taxonomic classification [10]. Given the immense size of sequencing datasets and the repetitive nature of this task, k-mer searching often represents a significant computational bottleneck. Moreover, when k-mer search involves sensitive personal or clinical genomic data [11], it introduces critical privacy concerns, especially when outsourcing computation to third-party servers or cloud infrastructures. Homomorphic encryption (HE) offers a promising solution to the challenge of secure computation on encrypted data [12]. HE schemes allow arithmetic operations to be performed directly on ciphertexts, enabling a server to compute over encrypted inputs and return encrypted results that can be decrypted only by the data owner. In the context of genomics, this means that sensitive sequence data and queries can remain encrypted throughout the computation, removing the need for trust in the computing infrastructure. There has been relevant work in the field, primarily [13], which introduced a privacy-preserving bit string search. While their method provides strong semantic security, it is limited to single k-mer queries, has a higher multiplicative depth due to its randomization step, and lacks support for parallelism. The foundational work by Kim and Lauter [14] demonstrated the feasibility of performing complex genomic computations, such as Hamming distance and chi-squared tests, using HE. Their protocol for Hamming distance cleverly uses Fermat’s Little Theorem to count mismatches but requires a high multiplicative depth, making it computationally intensive and limiting its practical throughput. Furthermore, their approach was designed for preprocessed genomic vectors and did not address the challenges of handling raw sequencing data, multi-k-mer scalability, or the biological need to filter results based on data quality. A different paradigm for collaborative analysis was introduced by Cho *et al*. [15], who demonstrated a scalable framework for distributed genome-wide association studies (GWAS) using secure multi-party computation (MPC). Their work enables multiple institutions to pool their computational resources, with the core operation being the secure calculation of statistical tests like the chi-squared statistic across their combined datasets without a trusted third party. However, MPC-based solutions are often difficult to deploy, in practice, as they require simultaneous online participation and complex coordination between all contributing parties. While pioneering for secure, large-scale statistical analysis, their framework is specialized for association testing and does not provide a mechanism for high-throughput k-mer searching within individual genomic sequences.

In this work, we present KmerCrypt, a framework for scalable and private exact k-mer searching on outsourced genomic data using HE. Designed to address the limitations of prior methods, our system is optimized for high-throughput analysis, natively supporting both assembled genomes in FASTA format and raw sequencing reads from FASTQ files. The protocol leverages a direct sum-of-squared-differences method, which minimizes the multiplicative depth of the homomorphic computation compared with general-purpose distance metrics, allowing for more performant cryptographic parameters. Our architecture is based on a practical single-client, single-server model allowing a client to offload data storage and computational intensive computations to a remote server, without compromising data privacy.

## Materials and methods

### Cryptographic preliminaries: the Brakerski–Gentry–Vaikuntanathan homomorphic encryption scheme

Our protocol is built upon the Brakerski–Gentry–Vaikuntanathan (BGV) fully HE scheme, a leveled integer-based cryptosystem that supports both addition and multiplication on encrypted data. In BGV, a fresh ciphertext *c* encrypting a plaintext message *m* can be conceptually represented as a pair of polynomials $\left({c}_0,{c}_1\right)$ such that:


$$ \mathrm{Decrypt}\left( sk,c\right)=\left({c}_0+{c}_1s\right)\operatorname{mod}\kern0.33em q=m+e, $$

where $s$ is the polynomial secret key, $q$ is the ciphertext modulus, and $e$ is a small noise term inherent to the encryption process. Homomorphic addition of two ciphertexts results in a ciphertext encrypting the sum of the plaintexts, with a new noise term equal to the sum of the original noises. Homomorphic multiplication, however, is more complex, resulting in a quadratic ciphertext that depends on ${s}^2$ and whose noise grows quadratically. To manage this complexity and control noise growth, a relinearization procedure is employed, which uses a publicly available evaluation key to transform the quadratic ciphertext back into a linear one of the original size, albeit with an increase in the underlying noise. A key feature of BGV is its support for SIMD (single instruction, multiple data) operations through a technique called batching, which leverages the Chinese Remainder Theorem. This allows a single ciphertext to be treated as a vector of thousands of plaintext “slots,” where any homomorphic operation is applied to all slots in parallel, providing a significant source of computational speed-up.

### Protocol implementation

**Figure 1:**
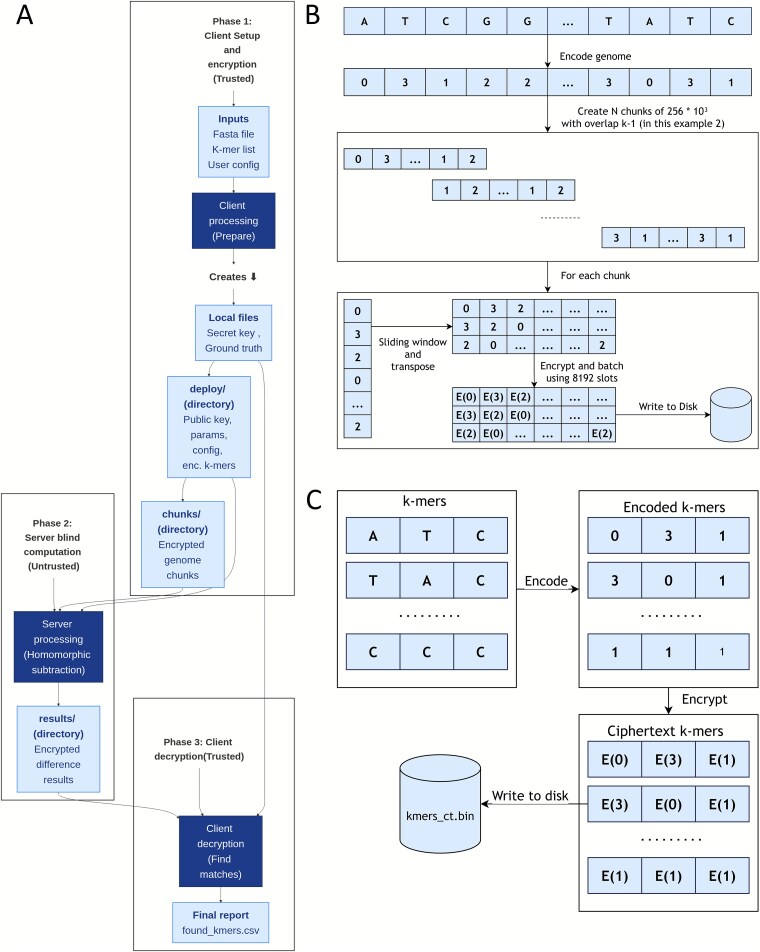
Protocol overview of kmerCrypt including genome and k-mer encryption process. (A) Security protocol of kmerCrypt illustrates involved parties, security parameters, data subjected to homomorphic computation, and resulting outputs. (B) Data transformation pipeline for the input genome in FASTA format, progressing from plaintext to encrypted genome chunks. FASTQ files including their quality scores are encrypted and processed in an equivalent manner. (C) Data transformation pipeline of k-mers from plaintext to on-disk ciphertext.

The system implements a private k-mer search protocol under an honest-but-curious server model, where the server is assumed to follow the protocol but may attempt to infer information from the data it processes. The cryptographic foundation is the BGV HE scheme, implemented using the Microsoft SEAL library. The Fully Homomorphic Encryption (FHE) parameters were configured to provide a 128-bit security level, utilizing a polynomial modulus degree of 8192, the Microsoft SEAL BFVDefault coefficient modulus for *n* = 8192, and a batching-friendly 20-bit plaintext modulus. All sensitive operations, including key management and data handling, are confined to a trusted client application, which communicates with the server via the filesystem (Fig. 1A).

The protocol’s design is optimized for the SIMD batching capabilities of the BGV scheme. During the client-side encryption phase, DNA bases {“A”, “C”, “G”, “T”} are encoded as integers {0, 1, 2, 3}, whereas padding and unknown characters are encoded with {4}. The target genome or set of sequencing reads is partitioned into independent chunks. For FASTA files, this involves creating overlapping segments of 256 KB. For FASTQ files, whole reads are batched together into chunks, and a padding of *k*_max_−1 non-nucleotide characters is inserted between concatenated reads to prevent the formation of erroneous k-mers at read boundaries. For each chunk, the sequence data are then transposed into *k*_max_ “stride” vectors, where each vector *j* contains the *j*th character of every possible k-mer window in that chunk for a *k* up to *k*_max_. These stride vectors are then encrypted into a set of *k*_max_ ciphertexts (Fig. 1B). Similarly, each of the *k* characters of a query k-mer is encrypted into a separate ciphertext where the character’s encoded value is replicated across all SIMD slots (Fig. 1C).


\begin{align*}& E\left(\mathrm{SSD}\right)\\&=\left\{\sum \limits_{j=0}^{k_{\mathrm{max}}-1}{\left(E\left({C}_{i+j}\right)-E\left({K}_j\right)\right)}^2\mathrm{for}\kern0.33em i=0,1\dots, \kern0.33em \mathrm{chunkSize}-{k}_{\mathrm{max}}-1\right\} \end{align*}

**Figure 2:**
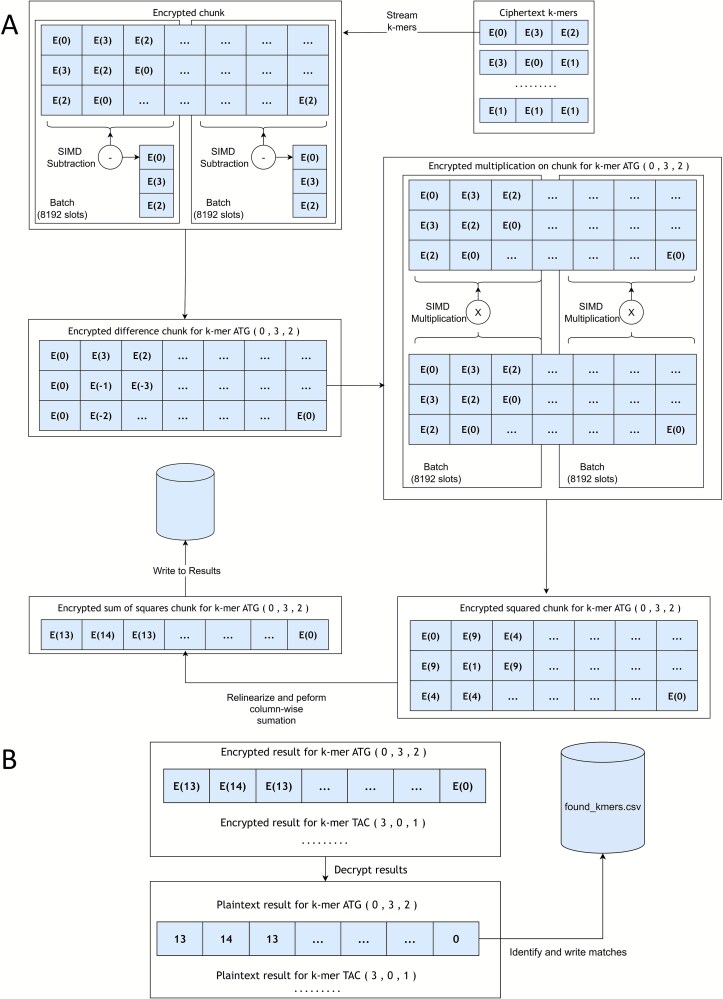
Server computation and client decryption. (A) Streaming of k-mers to execute homomorphic sum of squared differences on a single chunk within each batch of size 8192, employing SIMD instructions. The illustration depicts the first k-mer, with subsequent k-mers processed in a comparable manner. (B) Client-side decryption of encrypted results, showing decryption of one chunk, calculation of the Hamming distance per window, and recording of the results to an output file.

The core of the untrusted server’s computation is a homomorphic sum-of-squared-differences, SSD. For each query k-mer, the server iterates from *j* = 0 to *k*−1, homomorphically subtracting the *j*th encrypted k-mer character from the *j*th encrypted genome stride ciphertext for the strides that correspond to the k-mer length being searched. The resulting difference vector is squared via homomorphic multiplication and added to an accumulating sum ciphertext. After *k* iterations, the final ciphertext contains the sum of squared differences for every k-mer window in the chunk, computed in parallel. A match is indicated if and only if this sum is zero, a condition that occurs only when all character-wise differences are zero (Fig. 2A). So the match condition becomes:


$$ \mathrm{Match}(i)\iff E\left({\mathrm{SSD}}_i\right)=E(0) $$

In the final decryption phase, the client decrypts the result vectors from the server. It identifies a match at any slot containing the value zero, mapping this slot’s index back to its original genomic coordinate or read-specific position (Fig. 2B). For FASTQ data, the client then correlates these matches with their corresponding Phred quality scores from the original plaintext file. This enables a user-specified filtering step, where matches can be discarded if their associated bases do not meet a minimum, average, or sum quality score threshold. To ensure correctness, the client initially performs a plaintext search to generate a ground truth dataset, which is used to verify the final FHE results. The entire workflow is parallelized using OpenMP; the server processes multiple data chunks concurrently, and the client parallelizes both the initial encryption and final decryption stages to ensure efficient use of multi-core processors.

The system is implemented in C++17 as two executables: a client for trusted operations and a server for untrusted computation. Data are managed through a file-based interface where the client populates deploy and chunks directories, and the server writes to a results directory. The system includes performance monitoring to measure the duration of computational phases and report peak memory usage. The client’s command-line interface enables distinct operational modes, including an encryption stage to prepare data and a decryption stage to process results, which facilitates the separation of workflow steps.

### Noise management

The system employs the BFV HE scheme, configured for 128-bit security with a polynomial degree of 8192. It utilizes the standard coefficient modulus chain for this degree, totaling ~218 bits, and a 20-bit plaintext modulus.

A freshly encrypted ciphertext’s noise budget is primarily determined by the 218-bit coefficient modulus. The computational circuit, which calculates the sum of squared differences (subtract, square, and accumulate), has a multiplicative depth of one. In this process, the squaring operation and its subsequent relinearization are the main consumers of the noise budget, while additions have a negligible impact.

This was validated by monitoring the invariant noise budget during server evaluation. Empirically, the minimum observed budget after all computations was ~110 bits. This substantial remaining margin is more than sufficient to ensure correct decryption, allowing for reliable zero/non-zero detection of the final results. Furthermore, since the plaintext outputs are small integers (bounded by 16), they are well within the capacity of the 20-bit plaintext modulus, preventing any risk of overflow.

## Results

### Case study

A direct homomorphic evaluation of the position weight matrix (PWM) log-likelihood scoring method is computationally infeasible with current FHE schemes. The protocol would require a transition to a real-number-aware scheme such as Cheon-Kim-Kim-Song (CKKS), which is designed for approximate arithmetic on encrypted floating-point numbers [16], and more critically, would necessitate a “private value selection” operation for every character in every genomic window. To determine the score for an encrypted nucleotide, the server would need to homomorphically evaluate which of the four possible scores to select from the PSSM, a process that involves a cascade of expensive homomorphic multiplications and conditional logic. Finally, comparing the resulting encrypted score against a threshold would introduce another layer of high-overhead homomorphic operations. Executing this entire sequence for millions of sliding windows would render the direct approach impractical for genomes of any significant size.

To circumvent this infeasibility, our method employs a problem reduction strategy that transforms the probabilistic search into a deterministic one, beginning with a position probability matrix provided in the standard MEME format, similar in principle to the computation performed by tools like FIMO [17]. This method avoids the expensive problem of private value selection inherent to a direct homomorphic approach. The expense arises because determining the score for an encrypted nucleotide requires the server to simulate an index lookup, often by homomorphically computing:


$$ 1-{\left(E\left({s}_j\right)-E(b)\right)}^{p-1} $$

based on Fermat’s little theorem to test for equality with each k-mer base $b$. This operation involves a costly homomorphic exponentiation, a procedure known to have a computational cost that scales with the logarithm of the exponent and requires a large number of successive homomorphic multiplications [18]. Instead, we perform a one-time, offline precomputation that exhaustively enumerates all possible k-mers for the motif’s length. While this precomputation is exponential in the motif length, it remains practical for most bioinformatics applications, as transcription factor binding motifs typically fall within the 6–12 base pair range. This reduction, however, introduces known limitations. The statistical framework relies on *a priori* background nucleotide frequencies, as the client cannot scan the server’s private genome to determine its true nucleotide composition. This requires using a generalized model that may not accurately reflect the target, potentially skewing the significance of the results.

The reduction is implemented as a standalone utility that generates the final k-mer query set. This tool first constructs a PSSM from the input PWM and specified background frequencies. It then calculates the complete score distribution using dynamic programming, creating an efficient mapping from any score to a corresponding p-value. The core of the utility is a parallelized loop that iterates through all 4^k^ k-mers, calculates their PSSM score, and assigns a p-value from the precomputed distribution. Following this enumeration, the entire set of k-mer matches is sorted by p-value to perform a Benjamini–Hochberg correction, assigning a q-value to each. Finally, the utility filters this comprehensive list, writing out two files: a simple text file containing only the sequences of k-mers that meet the user-defined significance threshold, ready for use in the FHE protocol, and a detailed CSV file associating each of these significant k-mers with its corresponding score, p-value, and q-value for later analysis.

### Profiling

To characterize the computational resource allocation across our implementation, we profiled wall-clock time I/O and compute in each phase, expressing results as normalized percentages (Supplementary Fig. S1). We report the following breakdown: (i) Phase 1 (client setup/encryption): 15.7% I/O, 84.3% compute, (ii) Phase 2 (server evaluation): 2.3% I/O, 97.7% compute and (iii) Phase 3 (client decryption/aggregation): 4.0% I/O, 96.0% compute.

These results show compute dominates in all phases, with the server’s homomorphic evaluation most compute-bound; client phases are also compute-heavy, with modest I/O from chunking and result handling.

## Discussion

The objective of our KmerCrypt toolkit is to provide a practical and optimized FHE-based solution specifically for the high-throughput task of exact k-mer searching. The goal is not to establish performance superiority over non-private or symmetric cryptographic methods, which will always be faster, but rather to enable secure computation in a zero-trust, single-server model, where data confidentiality is paramount and multi-party coordination is not required. Our implementation introduces specific trade-offs; for instance, the novel Phred score filtering feature necessitates a data scanning step on the trusted client during decryption to correlate results with quality scores for the minimum phred score threshold version. This limitation is based on the fact that while it is straightforward to compute the sum of qualities server sided the minimum quality score requires non-linear aggregates which result in unpredictable and exploding multiplicative depth. To keep the approach in parity with the k-mer version, we only implement the sum and average threshold of qualities using the same method as with the k-mers, and we transfer the scanning cost to the client in the case of the minimum threshold. Furthermore, a deliberate tradeoff is made within the FHE design itself: our deterministic sum-of-squares protocol prioritizes a minimal multiplicative depth for higher performance, in contrast to randomized protocols that offer stronger resistance to ciphertext side-channel attacks at the cost of greater computational complexity. A limitation of the proposed approach is that genomes must be encrypted with respect to a fixed parameter *k*_max_, representing the maximum k-mer length to be queried. This strategy tradeoffs ciphertext size to enable users to query multiple k-mer lengths which we find essential for a real world application. This strategy though has two obvious limitations the first one being that the query k-mers are limited by *k*_max_ and the second being redundant storage in the case of smaller k-mer queries which are more common in real-world applications. We provide users the ability to tune this tradeoff based on their workloads via the *k*_max_ parameter.

A potential optimization to our approach would involve enabling the server to detect large, contiguous regions of the encrypted result vector that are free of matches. One method to achieve this is to homomorphically compute the product of all values within a large block of slots; if the product is non-zero, the entire block contains no matches and can be discarded. This approach, however, substantially increases the required multiplicative depth and is computationally expensive [19]. A more practical alternative leverages Fermat’s Little Theorem to implement a zero-test, where each slot is homomorphically mapped to 1 if it contains a non-zero value and to 0 otherwise. After aggregating these mapped values, a sum of zero indicates that the entire block is free of matches. While this strategy has a much lower multiplicative depth, it still incurs additional computational overhead on the server. Both techniques would allow the server to construct a coarse-grained index on the client’s behalf, reducing the volume of data that must be transmitted and decrypted. We leave the implementation of these optimizations as future work.

**Table:** 

Abbreviation	Description
*General and Bio. terms*	
BGV	Brakerski–Gentry–Vaikuntanathan
FHE	fully homomorphic encryption
GWAS	genome-wide association studies
HE	homomorphic encryption
MPC	secure multi-party computation
PPM	position probability matrix
PWM	position weight matrix
SIMD	single instruction, multiple data
SSD	sum-of-squared-differences
*Cryptographic Terms*	
c	ciphertext
m	plaintext message
e	noise term
q	ciphertext modulus
s	polynomial secret key
sk	secret key for decryption

**Key Points**

- KmerCrypt allows secure k-mer searches over encrypted genomic data by leveraging homomorphic encryption, keeping sensitive information confidential at all times.
- The protocol supports high-throughput queries in both FASTA and FASTQ formats, enabling practical privacy-preserving bioinformatics analyses in cloud environments.
- Implementation minimizes cryptographic overhead via Single Instruction, Multiple Data batching and low multiplicative depth, ensuring results are decryptable by the data owner only.

## Supplementary Material

Supplementary_Material_kmer_crypt_bbaf648

## Data Availability

All relevant code and results can be found on GitHub at this link: https://github.com/Georgakopoulos-Soares-lab/kmer_crypt.
