# Supplementary material for: KmerCrypt: private k-mer search with homomorphic encryption
Source: Brief Bioinform. 2025 Dec 8;26(6):bbaf648. doi: 10.1093/bib/bbaf648 (PMC12684705; doi:10.1093/bib/bbaf648)
Supplement: Supplementary_Material_kmer_crypt_bbaf648 [file supplementary_material_kmer_crypt_bbaf648.pdf]

Supplementary Material

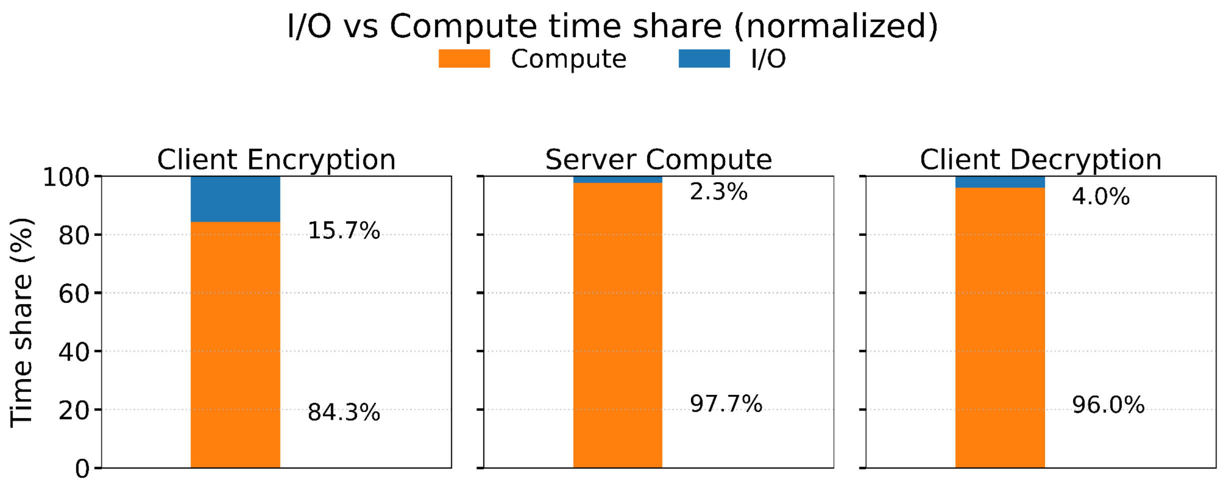

Supplementary Figure 1: Percent of total time shared in compute and input/output (I/O). Results shown for Client Encryption, Server Compute and Client Decryption.
